# VR-based nature interventions for health promotion in long-term care facilities (Nature Boost)—a mixed-methods feasibility study protocol

**DOI:** 10.3389/fdgth.2026.1727618

**Published:** 2026-07-16

**Authors:** Nele Sieverding, Judith Czakert, Steven Ngandeu Schepanski, Wiebke Stritter, Georg Seifert

**Affiliations:** 1 Charité Competence Center for Traditional and Integrative Medicine (CCCTIM), Charité - University Medical Center Berlin, Corporate Member of Freie Universität Berlin and Humboldt-Universität zu Berlin, Berlin, Germany; 2 Department of Pediatrics, Division of Oncology and Hematology, Charité - University Medical Center Berlin, Corporate Member of Freie Universität Berlin and Humboldt-Universität zu Berlin, Berlin, Germany

**Keywords:** aging, feasibility study, health promotion, long-term care, mixed methods, nature interventions, nursing staff, virtual reality

## Abstract

**Aim:**

To assess the feasibility and acceptability of a virtual reality-based nature intervention in long-term care facilities and explore psychosocial effects ahead of a future randomised controlled trial.

**Design:**

Two-centre feasibility study in a mixed-methods design, focusing on qualitative evaluation.

**Methods:**

Ten residents and ten nursing staff members from two long-term care facilities complete twelve 15-minute virtual reality sessions using a 360° forest video developed by the study team. Over four weeks, residents conduct three sessions each week. Six residents receive support by nursing staff once weekly. The primary outcome feasibility will be qualitatively assessed through semi-structured interviews and qualitative content analysis. Secondary outcomes, including sleep quality, anxiety, depressive symptoms, wellbeing, loneliness and trust in nursing staff, will be analysed using quantitative descriptive analysis.

**Conclusion:**

The study will provide insights into the feasibility of implementing virtual reality-based nature interventions in long-term care, informing a full-scale effectiveness trial.

**Impact:**

The feasibility study will provide information about the barriers and facilitators of the VR-based nature intervention for long-term care facility residents and nursing staff. This information will offer valuable insights into the feasibility of VR interventions in long-term care settings for (workplace) health promotion. Information about the potential effectiveness of the Nature-Boost VR intervention will offers useful perspectives for adapting and improving similar interventions.

**Reporting method:**

This protocol (Version 1.0) complies with the SPIRIT-Outcomes 2022 and TIDieR checklist.

**Patient and public involvement:**

Residents and nursing staff contribute feedback to refine the intervention.

**Clinical Trial Registration:**

https://drks.de/search/en/trial/DRKS00037568/details, identifier DRKS00037568.

## What does this paper contribute to the wider global clinical community?

- This protocol introduces immersive virtual nature into everyday care routines, offering a low-barrier, relationship-focused mental health intervention for populations with limited access to natural environment (e.g., geriatrics, palliative care, neurorehabilitation).
- By fostering trust and strengthening nurse-patient relationships, the intervention addresses a key global challenge in long-term care: maintaining quality relationships despite staff and time constraints.
- The outlined feasibility framework provides a template for international adaptation of virtual nature interventions across diverse healthcare systems.
- It challenges the clinical community to reimagine the therapeutic landscape of long-term care by merging cutting-edge technology with deeply human needs: connection, calmness, and presence.

## 1 Introduction

The United Nations estimates the global population aged 65 years and older will more than double from 727 million in 2020 to 1.5 billion by 2050, accounting for 16.0% of the global population (1). Meanwhile, according to the World Health Organization (WHO), there will already be a shortage of approximately 4.5 million nurses in 2030 (2). As a result, the number of people requiring long-term care is likely to rise, while the number of available care workers is declining, widening the gap between care needs and personnel resources. This imbalance increasingly limits the time available for individual attention and contributes to higher levels of stress and increased burnout rates among staff (3).

This development may also affect the residents of long-term care facilities, as the achievable quality of care depends, among other things, on the nurse-to-patient-ratio and the amount of time nursing staff can dedicate to each resident (4). Simultaneously, many residents have extensive care needs related to activities of daily living due to advanced age, limited mobility and complex geriatric conditions (5). As a result, residents may experience fewer social interactions and reduced sensory stimulation, which in turn increases the risk of affective impairments, loneliness, cognitive decline, and sleep disorders. Sleep disturbances can further heighten the risk of cognitive deterioration and increase mortality risk, for example through an increased risk of falls and strokes (6–9).

Beyond basic care provision, the quality of the nurse-patient relationship plays a critical role in residents' overall well-being. However, under conditions of staff shortages, it becomes increasingly difficult to build and maintain stable, trust-based relationships, largely due to limited time and attention (10). A strong nurse-patient relationship has been associated with improved physical and emotional well-being in care-recipients (11–13). Trust is often described both as prerequisite for and an outcome of such a strong nurse-patient relationship (14).

These developments underline the need for feasible, preventative and health promoting interventions that can be integrated into everyday life and care to support both residents and nursing staff under structural constraints. Within this context, nature-based interventions offer a promising candidate approach, as they combine low-threshold exposure to restorative environments with potential psychological and physiological benefits. Nature-based interventions, such as forest therapy and forest bathing [Shinrin-yoku, introduced by Tomohide Akiyama in Japan in 1982 (15)], have been associated with psychological restoration and improved (mental) wellbeing (16, 17). Further studies (18–22) suggest reductions in mental disorder, depressive, anxiety, and stress-related symptoms following exposure to natural environments. Some studies have also reported physiological indicators of relaxation, such as changes in blood pressure, cortisol, or prefrontal activity (23). This evidence provides a broader rationale for the restorative potential of nature exposure.

The Attention Restoration Theory posits that modern life demands, such as sustained focus at work, can lead to mental fatigue through overuse of directed attention. In contrast, nature-based interventions may engage involuntary attention through soft fascination, allowing the brain's executive system to rest and recover (24). A randomised controlled trial (RCT) by McDonnell & Strayer supported this theory by showing that nature walks lowered frontal midline theta activity in EEG which was interpreted as reflecting restoration of executive attention (25). Similarly, a systematic review by Song et al. (2016) found that nature-based interventions lowered the absolute haemoglobin concentrations in the prefrontal cortex, indicating relaxation (19). Notably, even isolated sensory stimuli, such as the scent of Hinoki cypress leaf oil, have been shown to elicit comparable effects (26). Hence, Attention Restoration Theory provides one theoretical explanation for why natural environments may support psychological restoration.

However, for many individuals with limited mobility, such as residents in long-term care facilities, direct access to natural environments is often not feasible (27). In such cases, technological alternatives may offer partial access to nature's sensory stimuli. Studies have shown that even viewing photographs or videos of natural landscapes, especially forests, can induce physiological relaxation by reducing sympathetic nervous system activity (28, 29). Additionally, a decrease in perceived loneliness was reported in an online survey conducted during the COVID-19 pandemic after participants watched nature videos (30). Building on these findings, digital nature interventions using virtual reality (VR) offer an even more immersive experience by using realistic, customisable 360° videos, providing nature experiences that can be tailored to individual preferences, without the need for physical relocation. Both healthy individuals and those with mental health conditions have shown improvements in stress, anxiety, and depressive symptoms following exposure to this form of digital nature-based intervention (31, 32).

The present feasibility study focuses on psychosocial and relational domains that are highly relevant for residents in long-term care. Wellbeing, loneliness, affective symptoms, and sleep quality are closely interconnected in older adults. These outcomes are influenced by social isolation, reduced social participation, mobility limitations, and environmental factors that restrict opportunities for meaningful engagement and stimulation (33–35). VR-based nature exposure may provide a low-threshold opportunity for distraction, soft fascination, relaxation, and positive emotional engagement within the care setting (32, 36, 37). In addition, when sessions are conducted jointly with nursing staff, the shared experience may provide an opportunity for interpersonal connection and may enhance the nurse-patient relationship and the residents' perceived trust in nursing staff. In addition, when sessions are conducted jointly with nursing staff, the shared experience may provide an opportunity for interpersonal connection and may enhance the nurse-patient relationship and the residents' perceived trust in nursing staff.

In the present feasibility study, the Attention Restoration Theory informs the conceptual rationale for using immersive VR-based forest exposure in long-term care. The primary goal of VR-based nature interventions is to provide visual nature experiences to long-term care residents despite mobility limitations, thereby supporting their well-being and potentially preventing outcomes such as sleep disturbances, affective disorders, loneliness, and reduced trust in nursing staff. The study focuses on feasibility, acceptability, and implementation in routine care, while exploratory questionnaire data will describe changes in sleep quality, anxiety and depressive symptoms, subjective wellbeing, loneliness, and trust in nursing staff.

**Figure 1:**
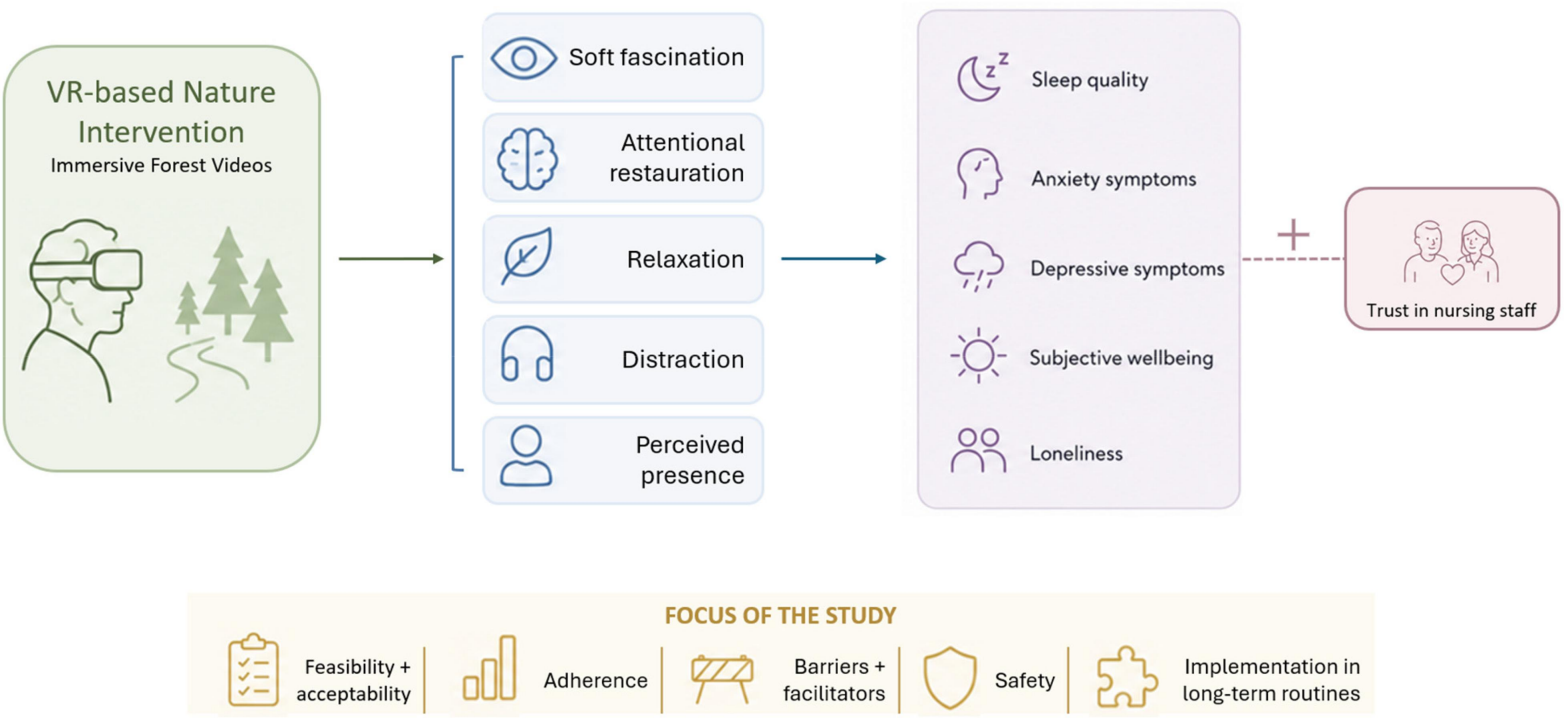
Conceptual framework of the nature boost feasibility study.

Figure 1 illustrates the assumed pathways of the VR-based nature intervention. The immersive forest videos are expected to provide a restorative nature experience through soft fascination, attentional restoration, relaxation, distraction, and perceived presence. These processes may contribute to exploratory changes in sleep quality, anxiety and depressive symptoms, subjective wellbeing, and loneliness. In joint sessions with nursing staff, the shared VR experience may additionally support interpersonal connection and trust in nursing staff. The primary focus of the present study is feasibility and acceptability, including adherence, barriers and facilitators, safety, and implementation within long-term care routines.

*Nature Boost* aims to examine the acceptance, feasibility, influencing factors and potential barriers of VR-based nature interventions in long-term care facilities in Germany. Given that older adults often have limited experience with VR technology, this preliminary evaluation is crucial. Identifying any challenges in implementation will help optimise the design of the subsequent RCT, which aims to assess the intervention's effectiveness.

## 2 The study

The overall aim of the study is to assess the feasibility and acceptance of a VR-based nature intervention in long-term care facilities from the perspectives of nursing staff and residents, and to explore potential effects on health and well-being.

### 2.1 Primary objective

To evaluate the feasibility and acceptability of implementing a VR-based nature intervention in long-term care facilities from the perspectives of residents and nursing staff.

### 2.2 Secondary objectives

- To identify barriers to implementation from the perspectives of residents and nursing staff.
- To assess changes in the nurse-patient relationships resulting from joint sessions.
- To explore potential effects of the intervention on sleep quality, mood, subjective well-being, feelings of loneliness among residents, and trust in nursing staff.

These outcomes address challenges that are especially common among long-term care residents and have previously been shown to benefit from nature-based interventions in prior studies (18, 20).

### 2.3 Research questions and exploratory objectives

The feasibility study *Nature Boost* will be conducted using a parallel mixed-methods design with a qualitative focus (38). The exploratory research design gives rise to the following qualitative research questions:

- How is the use of VR-based nature interventions in long-term care facilities perceived by residents and nursing staff in terms of acceptance and feasibility?
- What specific barriers to implementation are reported by residents and nursing staff?
- How does the interventions shape the nurse-patient relationship?
- What additional potentials are reported in the interviews by residents and nursing staff?

For the quantitative strand of the study, the following exploratory research question is formulated: Can descriptive changes in sleep quality, anxiety and depressive symptoms, subjective well-being, loneliness, and trust in nursing staff be observed over the course of the VR-based nature intervention? Due to the exploratory nature of the feasibility study, no fixed efficacy endpoints are defined. Instead, the qualitative research questions and exploratory quantitative objective will be investigated in an open-ended manner to inform the design of a future effectiveness trial.

## 3 Methods

**Figure 2:**
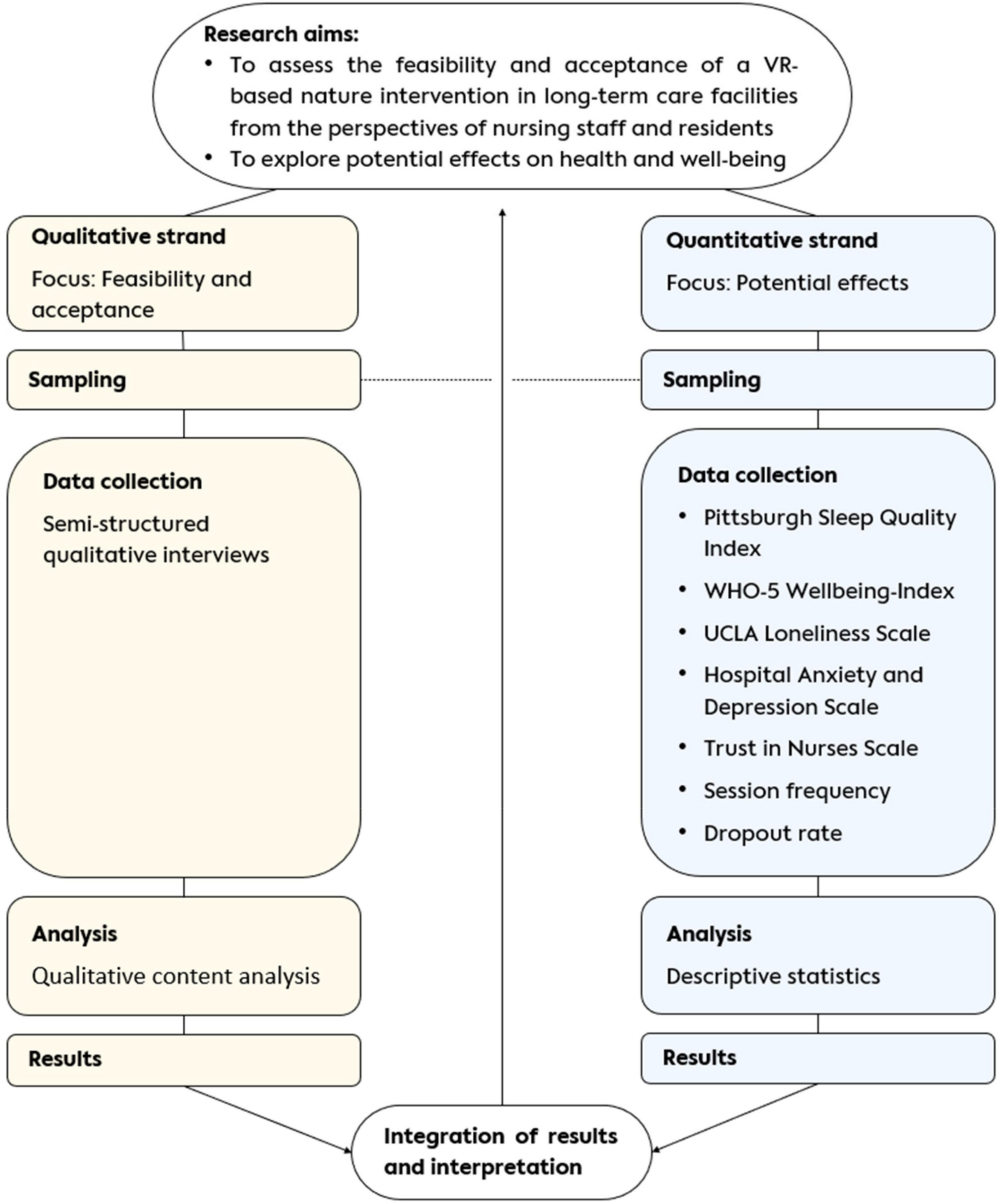
Study design overview. A parallel mixed methods design with qualitative focus is implemented. Orange boxes mark the qualitative, blue boxes the quantitative research strand. The dotted line represents the overlap of both strands during sampling. Semi-structured qualitative interviews form the qualitative, questionnaires the quantitative assessment. Integration of both strands will be conducted in the interpretation phase to enable a comprehensive exploration of the research aim. Illustration based on Kuckartz (39).

The pilot study employs a parallel mixed-methods design (see Figure 2), with a focus on the qualitative component (38). This mixed-methods approach was chosen to explore both the participants' subjective experiences, the feasibility of the intervention, and to get first insights into potential effects of the intervention. The quantitative component will be used to assess the feasibility, completeness, participant burden, and suitability of the selected outcome measures. Descriptive changes in questionnaire outcomes will be interpreted cautiously and used to identify promising outcome domains for a future effectiveness trial. The design of a subsequent RCT will additionally be informed by feasibility parameters from the present study, clinically meaningful target differences, and evidence from the wider literature. The combination of qualitative and quantitative research strands allows the exploration of contextual factors and barriers that may arise during the following RCT and are not captured through quantitative data alone. Specifically, the impact of the implementation in different long-term care facilities and if residents are able to conduct sessions independently will be analysed.

### 3.1 Setting

To identify contextual factors, the intervention will be implemented in two long-term care facilities in Berlin, Germany. The selection of two facilities from different socioeconomic areas is intended to capture variation in implementation context. Facility-level socioeconomic context may influence feasibility through several pathways, including differences in institutional resources, staffing routines, residents' previous exposure to digital technologies or nature-based activities, health literacy, mobility limitations, and the amount of support required during the VR sessions. These contextual factors may affect how acceptable, accessible, and practical the intervention is in everyday care and will therefore be explored qualitatively as potential influences on implementation.

### 3.2 Intervention

In both facilities, participants engage with immersive 360° forest videos, developed by the research team. The videos will be shown using Meta Quest 3S VR headsets (non-medical device). The forest shown in the videos represents a typical German mixed woodland and is intended to evoke a familiar environment for participants. Twelve different videos of forest walks were produced. Participants watch three 15-minute videos per week over a four-week period. They can freely look around during each session.

The VR intervention was developed by the research team to provide a low-threshold audiovisual approximation of a forest walk for residents with limited access to natural environments. The videos were filmed during walks through Schorfheide-Chorin UNESCO Biosphere Reserve in autumn using the INSTA360 X3 Actioncam 360° camera. Each video consists of a continuous recording without cuts to preserve the perceived flow of walking through the forest. The camera operator walked at a slow and steady pace intended to be suitable for older adults. The camera was positioned at approximately 180 cm. Audio was captured using the camera's built-in microphone and consists of natural ambient forest sounds, including wind, birdsong, and footsteps on the forest floor.

The intervention engages participants primarily through visual-spatial immersion and auditory stimulation. Participants can freely look around during the 360° video, creating a sense of presence within the forest environment. Real-world forest exposure remains a broader multisensory experience, and future adaptations may explore additional sensory components.

Before implementation in the long-term care facilities, the videos and technical procedures were reviewed by members of the research team as well as two external research associates with experience in nature-based interventions and two students not affiliated with the project and without former VR experience. This preliminary testing focused on technical functionality, usability, tolerability, perceived image and sound quality, and the suitability of video duration and pacing. Feedback from this phase was used to refine the video selection and implementation procedures before use in the real-world care setting.

Five out of 10 residents will complete one VR session per week together with a member of the nursing staff^1^. The participating staff member may vary between sessions depending on shift schedules, workload, and availability. This approach reflects real-world long-term care routines, where residents are commonly supported by several members of the nursing team. Accordingly, trust is conceptualized as residents' perceived trust in nursing staff more broadly. The qualitative interviews will explore how residents and nursing staff perceive the joint sessions, including whether shared VR experiences influence interpersonal connection, perceived support, and trust in nursing staff under routine care conditions.

The remaining four residents will conduct the sessions independently following an initial briefing and instructions from nursing staff. Residents receive a personal VR headset for the duration of the intervention and complete the sessions before bedtime in an upright position in bed or another seated position.

The VR system is preconfigured to minimise handling steps. Specifically, the VR headsets will be fully configured by the research team before use. Residents are not expected to navigate the standard Meta Quest menu or select videos independently. Instead, the respective intervention video will be preselected and started by nursing staff after instruction. Thus, residents only need to wear the headset and experience the video, while technical handling is kept to a minimum. For joint sessions, nursing staff will use shared VR headsets, which require a short calibration phase before each session.

At the beginning of the intervention period, an information session with hands-on training is held with participating residents and nursing staff to explain the use of the VR headsets, outline the course of the intervention, and address any questions. Usual care and daily routines within the long-term care facilities, regular medication, therapeutic procedures and social activities may continue unchanged during the intervention period.

After each use, the VR headsets and contact surfaces will be cleaned and disinfected according to the facility's hygiene standards.

After each session, the intervention will be documented in an individual intervention diary, noting the session date, duration, whether the session took place individually or in a resident-nursing staff dyad, whether a nursing staff member was present, and any relevant comments. This diary is developed and provided by the research team to help track the sessions and will be explained to participants prior to the intervention.

If participants are not able to complete a session, the next session will be supported by a nursing staff member, including a repetition of the technical instructions if needed. The reason for the missed or incomplete session should be documented in the intervention diary. The intervention was described according to the TIDieR checklist (Appendix).

### 3.3 Sampling

A total of 20 participants, including residents (*n* = 10) and nursing staff (*n* = 10) who meet the inclusion criteria (see Table 1), will be recruited from the two long-term care facilities. Given the exploratory study design, no formal sample size calculation was conducted, as this sample size is expected to provide sufficient depth of data and a broad range of perspectives to explore the key feasibility objectives. We retain the option to expand the sampling at a later stage, if needed.

**Table 1:** Inclusion and exclusion criteria.

Inclusion criteria	Exclusion criteria
Residents	
Leaves care home ≤1x per week	Contraindications to the use of VR technology (severe balance disorders, epilepsy)
Living in a long-term care facility at least for the time of the intervention and interviews	Participation in a similar study within 12 months
Sufficient physical and mental ability to participate in the intervention [turn the head to explore the VR-simulation, independent (dis-) mounting of the headset]	Physical or mental conditions that impair participation in the intervention or data collection (mild & severe cognitive deficit, not compensable severe visual or auditory impairments)
	Impaired ability to give consent due to physical or mental limitations
	Medical devices sensitive to magnetic fields (pacemakers, defibrillators, special hearing aids and VP shunts)
	Severe psychiatric disorder (psychotic disorder, bipolar disorder)
Nursing staff	
German language proficiency of at least B2^a^	Contraindication to the use of VR technology (severe balance disorders, epilepsy)
	Participation in a similar study within 12 months
	Time-related limitations that interfere with study participation (e.g., planned holiday, shift plan)

Participation in similar study that exclude from this study are other virtual reality-based interventions or structured psychological programs that may influence mood, sleep, well-being, anxiety or depression.

a According to the Common European Framework of Reference for Languages (CEFR).

Recruitment will be initiated through gatekeeper, such as care facility management and staff representatives, who will be contacted via email, followed by a phone call or an in-person meeting to introduce the study. Informational flyers will be distributed and displayed within the facilities. The recruitment of residents and nursing staff will take place in close coordination with facility management and nursing staff, who propose eligible participants based on the eligibility criteria. Nursing staff may also volunteer to participate in the study.

**Figure 3:**
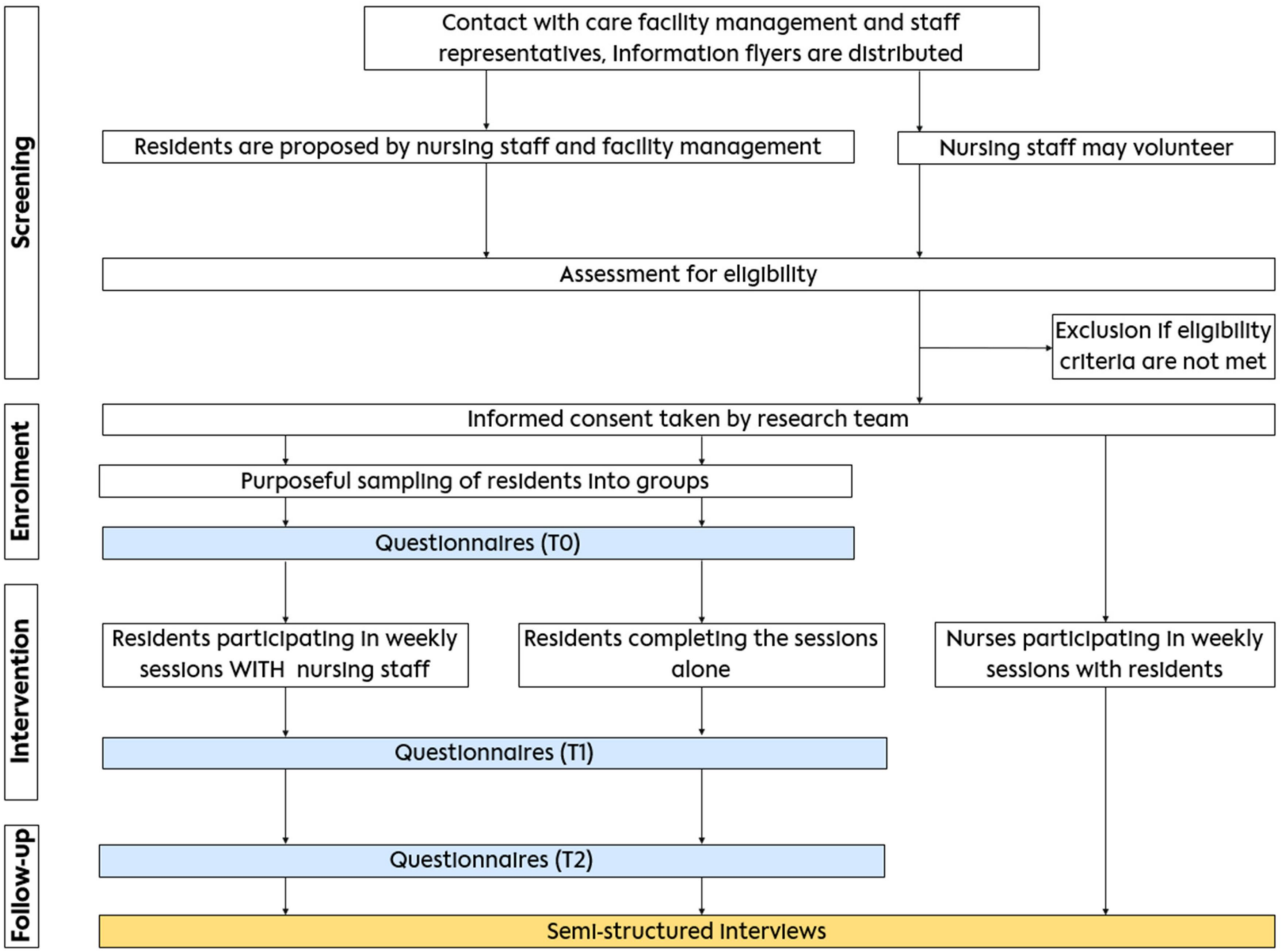
Participant timeline. The inclusion to the study includes an eligibility screening, the informed consent to participate and the qualitative purposeful sampling to intervention groups. Blue boxes mark quantitative and orange boxes qualitative assessment. The time points for data collection are: before the start of the intervention (T0), 2 weeks after the start (T1) and after the end of the intervention (T2).

For an overview of the participant timeline, see Figure 3.

All participants will be selected according to maximal variation sampling criteria to ensure a broad perspective on the research questions (40).

Maximal variation sampling will be applied pragmatically within the eligible pool available in the two participating facilities. The aim is to include a range of perspectives that are relevant for assessing feasibility and acceptability in a real-world long-term care setting. For residents, variation will be sought with regard to age, gender, and degree of mobility. For nursing staff, variation will be sought with regard to age, gender, and work experience. The exact number and socio-demographic composition of eligible residents and staff will depend on the participating facilities and will be documented during recruitment. Basic information on the available recruitment pool and the socio-demographic characteristics of included participants will be reported in the feasibility study.

Afterwards, residents will be assigned into two groups, aiming for an equal distribution of characteristics:

(1) “Residents completing a weekly VR session together with a nurse” and
(2) “Residents conducting the sessions independently after initial instruction”.

In the joint sessions, nursing staff actively participate by watching a 360° video with the VR headset together with the resident. In addition, they may provide support if the resident encounters difficulties with the device or procedure. Including participants from two separate facilities as well as the variation between individual and joint sessions with nursing staff allows the exploration of contextual variability. The sample is adequate to inform adjustments to the study design and intervention for a subsequent RCT.

### 3.4 Data collection

Data collection will be conducted on site at each pilot facility. Questionnaires will be administered at three time points: baseline (T0), two weeks after the start of the intervention (T1), and after completing the intervention (T2). Semi-structured interviews will be conducted post-intervention phase (T2) in a quiet, one-on-one setting to ensure privacy and minimise disturbances.

At baseline, basic socio-demographic and health-related characteristics will be collected to describe the sample and contextualize feasibility findings. For residents, these will include age, gender, duration of residence in the long-term care facility, degree of mobility, frequency of leaving the facility, relevant sensory limitations, previous experience with VR or similar digital technologies, and selected health information relevant to participation, such as contraindications to VR use and relevant physical or cognitive limitations. For nursing staff, baseline characteristics will include age, gender, professional role, years of professional experience, duration of employment in the facility, and previous experience with VR or similar digital technologies.

#### 3.4.1 Qualitative data collection

At T2, semi-structured qualitative interviews (41) will be conducted with all participants to explore acceptance and feasibility, potential effects on residents and the nurse-patient-relationship and challenges of the intervention. The interview guide will include open-ended questions on the experience of using the VR headset, perceived facilitators and barriers, technical and organizational challenges, and the integration of the intervention into daily care routines. To connect the qualitative and quantitative strands, residents will also be asked about perceived changes in sleep, mood, wellbeing, loneliness or social connection, and trust in nursing staff. Nursing staff will be asked about their own experience of the intervention, perceived changes in residents during or after the sessions, feasibility within routine care, and perceived effects on interaction and relationship with residents. Pilot interviews will be conducted to ensure that the interview guideline is adjusted to participants and suitable for exploring the research questions. Minor adjustments may be made.

The interviewer is trained in qualitative research methods and supported by a methodological experienced team.

#### 3.4.2 Quantitative data collection

In addition to interviews, residents will complete standardised questionnaires at T0, T1, and T2 to assess sleep quality, anxiety and depressive symptoms, psychological well-being, loneliness, and trust in nursing staff. All questionnaires will be verified onsite for completeness, and the data will be entered into a secure database.

Questionnaires used:

##### 3.4.2.1 Pittsburgh sleep quality index (PSQI)

To evaluate sleep quality, the Pittsburgh Sleep Quality Index will be administered (42). 19 self-rated questions provide information about seven components commonly affected in people with sleeping disorders (subjective sleep quality, sleep latency, sleep duration, habitual sleep efficiency, sleep disturbances, use of sleeping medication and daytime dysfunction). Each component is scored from 0 to 3, yielding a global score of 0 to 21. Higher scores indicate poorer sleeping quality, with scores >5 suggesting clinically relevant sleep problems. The validated German version has demonstrated acceptable reliability (Cronbach's *α* = 0.75) and validity and has been widely tested in elderly patients (43).

##### 3.4.2.2 The world health organisation-five well-being index (WHO-5)

The WHO-5 is a worldwide used questionnaire, consisting of five questions to evaluate subjective well-being in the past two weeks (44). Items are rated in a 6-point Likert scale from 0 (“at no time”) to 5 (“all of the time”), resulting in a raw total score of 0 to 25. For interpretation, scores are multiplied by four to yield a final score between 0 (the worst possible well-being) to 100 (the best possible well-being). A cut-off score of ≤50 is recommended to screen for depression.

The German version has shown excellent internal consistency (Cronbach's *α* = 0.92), strong construct validity, high acceptance and has been tested in older adults (45).

##### 3.4.2.3 UCLA loneliness scale—six item version (ULS-6)

The ULS-6 is a validated short version of the UCLA Loneliness Scale (46). It consists of 6 items to evaluate perceived loneliness, rated on a 4-point Likert-scale from 1 (“never”) to 4 (“often”), resulting in a score between 6 and 24 points. Higher scores indicate greater loneliness.

While the original 20-item version has shown high internal consistency (Coefficient *α* = 0.96) and great validity, a large cross-cultural study with participants from Germany, Indonesia and the USA found that it did not meet criteria for measurement invariance across different cultures (47). Therefore, the 6-item version (ULS-6) was proposed as it demonstrated configural, metric, scalar and error invariance, making it a reliable instrument for cross-cultural measurement of loneliness.

##### 3.4.2.4 Hospital anxiety and depression scale (HADS)

Symptoms of anxiety and depression and their severity will be assessed using the Hospital Anxiety and Depression Scale (HADS) (48). It consists of 14 items, with seven items concerning depression (HADS-D) and the other seven items concerning anxiety (HADS-A). Each statement is answered on a 4-point scale, with the self-descriptive response option varying from question to question. Possible answers inquire about the frequency or severity of symptoms or the extent of behavioral change from the past to the present. Two sum scales result that range from 0 to 21 points, with higher scores indicating more severe depression or anxiety and a cut-of ≥8 indicating clinically relevant symptoms. All items can be summed up to a total score from 0 to 42 for a global screening of mental illnesses, with a cut-off of ≥14 indicating clinically relevant mental health problems.

The validated German version has proven good internal reliability (Cronbach's *α* = 0.83 for HADS-A and 0.82 for HADS-D and 0.82−0.90 for the global scale), along with a solid content, factor and construct validity (49).

##### 3.4.2.5 Trust in nurses scale—4-item version

To measure the trust of patients in their treating nursing staff, the Trust in Nurses Scale 4-item version will be applied, with four questions regarding activities of nursing staff or feelings of patients (50). These are ranked on a 6-point Likert-type scale from 1 (“never”) to 6 (“always”). Scores are summed up and higher scores indicate higher trust in nursing staff. It demonstrated good internal consistency (Coefficient *α* = 0.82) and validity with clear factor structure and excellent model fit.

The original 5-point version will not be used in this study as the additional question addresses nursing staff communicating accurate information about the patients' cancer, which does not to the present feasibility study.

At the time of protocol development, no validated German version was available. Still, trust in nursing staff as indicator for the nurse-patient-relationship represents a key research objective in this feasibility study. Therefore, the original version was translated into German, following selected steps of the cross-cultural adaption process by Beaton et al. (51). Specifically, the questionnaire was initially translated (stage 1) by the author, a native German speaker. The review by a native speaker (Stage 3) was conducted by a native English speaker of the working group to improve linguistic clarity and conceptual consistency. Additionally, the translated version was reviewed by the writers of the original protocol draft with expertise in clinical psychology, research and nursing. Accordingly, this German version should be considered a preliminary translation, as no full cross-cultural validation with back-translation (stage 2), expert committee review (stage 4) and pretesting (stage 5) were conducted.

##### 3.4.2.6 Intervention diary

Moreover, a self-developed intervention diary will document session frequency and duration, whether sessions were conducted independently or with nursing staff, and any technical difficulties, interruptions or early termination. To capture immediate perceived responses to individual VR sessions with minimal participant burden, will rate their overall session experience on a 5-point scale ranging from 1 (“very unpleasant/not acceptable”) to 5 (“very pleasant/very acceptable”) and may note spontaneous impressions or notable experiences directly after the session. In addition, the overall dropout rate will be recorded.

### 3.5 Data analysis

#### 3.5.1 Qualitative data analysis

Data from interviews will be analysed with an inductive-deductive qualitative content analysis based on Kuckartz & Rädiker (52). Data will be managed and analysed with the qualitative data analysis software MAXQDA 24 (53). This allows for the structured development of categories to identify themes relevant to participants in terms of feasibility, acceptability and preliminary effects.

#### 3.5.2 Quantitative data analysis

Changes from baseline to T1 and T2 will be analyzed using descriptive statistics such as means, standard deviations, medians, frequencies, and ranges using SPSS (54). Descriptive summaries will be presented for the total sample and, where meaningful, separately by study site to explore whether feasibility indicators, missing data, adherence, or questionnaire trends vary between the two long-term care facilities. Site-specific descriptive patterns will be interpreted cautiously and considered alongside qualitative findings on contextual factors, barriers, and facilitators.

The analysis population will include all participants with data available at each respective time point, including those who discontinue the intervention prematurely. Missing data will be reported, but no imputation will be applied due to the small sample size. Given the exploratory nature of the study, no further statistical testing is planned at this stage. Session frequency and duration, as well as dropout rates, will be analysed descriptively to assess intervention adherence. Session ratings and spontaneous comments documented in the intervention diary will be analyzed descriptively and used to complement the qualitative interview data on acceptability, perceived short-term responses, and implementation feasibility.

No comparator is included in this feasibility study as the primary aim is to explore the feasibility of VR-based nature interventions in long-term care settings and not the efficacy. As this is a feasibility study with a small, exploratory sample size, no minimal important change is defined for individual participants and continuous outcome data will not be dichotomized or categorized for inferential analysis. Additionally, no composite outcomes are included in this study.

#### 3.5.3 Interpretation of qualitative and quantitative results

Integration of qualitative and quantitative results will occur during the interpretation phase using a convergent comparison approach. Descriptive trends in questionnaire outcomes, including sleep quality, anxiety and depressive symptoms, subjective wellbeing, loneliness, and trust in nursing staff, will be compared with interview data addressing the corresponding domains. The integration will focus on convergence, complementarity, and divergence between both strands. For example, descriptive changes in questionnaire scores will be interpreted alongside participants' narratives about perceived sleep, mood, wellbeing, social connection, and trust in nursing staff. Qualitative findings on feasibility, acceptability, barriers, and facilitators will provide contextual information for interpreting quantitative trends, missing data, adherence, and participant burden. The research team will collaboratively participate in the integration process through team discussions and consensus-building. There are no pre-specified criteria on how to proceed with the following RCT, as the feasibility study focuses on the qualitative feedback of participants to enhance the design of a future effectiveness trial rather than quantitative data.

## 4 Discussion

This protocol outlines the mixed-methods feasibility study *Nature Boost* which aims to explore the feasibility, acceptability and potential effects of a VR-based nature intervention in the context of long-term care facilities. Residents in these settings are particularly vulnerable to mental health challenges, such as loneliness, poor sleep, or depression, yet they often face mobility-related barriers that limit access to nature-based interventions (6–9). In parallel, nursing staff encounter structural challenges such as staff shortages, high workloads and limited time per resident, which can compromise individualised care (10).

The proposed intervention addresses this dual challenge by offering an immersive, low-threshold digital format that can be embedded into existing care routines. It is distinct from earlier approaches in that it systematically examines both the resident's and the nursing staff's perspectives, including the feasibility of implementation in daily workflows and the potential to strengthen the nurse-patient relationship through shared VR sessions (55, 56). This dual focus, on the user experience and the structural embedding, is largely absent in previous studies and constitutes a core innovation of *Nature Boost*.

Prior research has demonstrated the general feasibility of VR use among older adults. For instance, Abd El Fatah et al. (2024) evaluated VR-based reminiscence therapy in this population and reported only very mild side effects, although their findings were based on a single questionnaire (57). In this study, sessions lasted 30 to 45 min though sessions over 30 min are not recommended, because higher durations of sessions increase the risk for cyber sickness (58). The present study builds on this work by adopting a more comprehensive and context-sensitive approach. Rather than a one-time application, the intervention consists of 12 short sessions over four weeks, simulating repeated exposure within the participant's daily routines compared to others (59, 60). This allows for a more ecologically valid assessment of feasibility and potential effects. Moreover, the study explicitly examines both resident and nursing staff perspectives to identify barriers to implementation, assess adherence under real-life conditions, and explore whether shared VR sessions can support interpersonal connection and trust in the nurse-patient relationship. To our knowledge, this relation aspect of VR-based interventions in long-term care has not been addressed in previous studies.

A strength of this feasibility study is the parallel mixed-methods design. The design allows an in-depth and complex understanding of acceptance, implementation barriers and potential effects of the intervention. Moreover, the study provides a potentially transferable template for VR-based nature interventions in different healthcare settings, such as geriatrics, palliative care and neurorehabilitation, given its reliance on short, low-intensity sessions, minimal technical demands, and its flexible integration into existing workflow. Its emphasis on shared experiences between patients and care providers may also offer added value in settings where interpersonal connection is central to health progression.

This protocol responds to the urgent need for innovative, low-threshold therapeutic strategies in long-term care, particularly for older adults with limited mobility and unmet psychosocial needs. By offering a digital, sensory intervention that can be embedded into existing care routines without requiring additional personnel, the study explores a scalable and resource-sensitive approach to support mental health. The findings will inform refinements for a future full-scale effectiveness trial and may pave the way for multi-sensory extensions, such as the integration of olfactory stimuli. Future research should also explore long-term effects and implementation feasibility across various healthcare contexts with different structural conditions.

## 5 Conclusion

This protocol outlines the methodological framework for a mixed-methods feasibility study evaluating a digital, VR-based nature intervention in long-term care settings. The study will respond to a growing need for scalable, low-threshold mental health strategies that can be implemented despite structural challenges such as limited mobility among residents and increasing pressure on nursing staff. By investigating feasibility, acceptability, and preliminary effects from both resident and staff perspectives, the study aims to identify practical and relational conditions that influence implementation success. The inclusion of repeated, brief VR sessions embedded in daily care routines, some conducted jointly with staff, also allows for a first exploration of how such interventions may impact the nurse-patient relationship, an aspect rarely addressed in prior research. The findings will inform the development of a full-scale effectiveness trial and may contribute to future adaptations of digital, nature-based interventions across healthcare settings. Ultimately, the study lays the groundwork for accessible, multisensory approaches to psychosocial support in aging populations.

### 5.1 Ethics and dissemination

The study has been approved by the Ethics Committee of Charité—Universitätsmedizin Berlin (EA4/126/25). Any significant protocol modifications (e.g., changes to eligibility criteria, outcomes or data collection procedures) will be submitted to the Ethics Committee of Charité for review and approval prior to implementation. Relevant changes will also be updated in the trial registration and if applicable, reported in future publications or participant communication materials. Informed consent by the participants of the study will be obtained by members of the research team. Residents will receive both verbal and written information about the study. Written informed consent will be obtained prior to participation. The written consent forms and all study related documents for residents and nursing staff were approved by the Ethics Committee.

### 5.2 Conflict of interest

The researchers responsible for the study declare no financial or other competing interests. This investigator-initiated trial is fully conducted and financed by the research team, with no external sponsor or funding body involved. All trial activities including study design, data collection, management, analysis, interpretation, manuscript preparation, and publication decisions are overseen exclusively by the core study team; no coordinating centre, steering committee, endpoint adjudication committee, or external data management team are involved.

### 5.3 Publication

The results of the feasibility study are planned to be published in a peer-reviewed journal and the German Clinical Trials Register (DRKS). In addition, findings will be presented at scientific conferences and will be reported to participating care facilities. There are no publication restrictions. Authorship will follow the International Committee of Medical Journal Editors (ICMJE) criteria, and no professional writers will be used.

### 5.4 Data privacy

All collected data will be treated with strict confidentiality and pseudonymized by assigning unique participant codes. The identification list will be stored separately on an encrypted server accessible only to authorized members of the research team. Audio recordings from the semi-structured qualitative interviews, will be transcribed using automated transcription software (f4) that pseudonymises personal identifiers, any audio segments containing identifying information will be muted (61). The software is compliant with the General Data Protection Regulation (GDPR). Pseudonymised transcripts will be stored securely on password-protected servers with limited access. All electronic data will be stored on encrypted, password-protected servers, compliant with the Requirements of the GDPR, the Federal Data Protection Act (BDSG) and medical confidentiality (62, 63). Access to identifying data is restricted to essential study personnel during data collection and analysis and further limited post-study to authorised auditors or for legally mandated inspections. There are no contractual agreements that restrict investigator access to the data. All data transfers will be encrypted to prevent unauthorized access. Audio recordings will be deleted after data analysis and publication, while pseudonymized transcripts will be archived securely for 10 years.

### 5.5 Monitoring and safety

Participation in the feasibility study Nature Boost may be discontinued due to intolerable adverse reactions (e.g., dizziness, nausea, disorientation), serious health events that pose a significant health risk or require hospitalisation and externally triggered safety concerns as a manufacturer-initiated technical recall of the VR headset. Also, participants may withdraw voluntarily at any time without providing a reason. All discontinuations will be carefully documented and affected participants will receive medical attention if needed. Early withdrawal from the study will have no negative consequences for participants. In cases of mild and isolated symptoms as dizziness, participants may be offered the option to resume participation after a short break, provided no medical concerns persist and they explicitly consent. Any withdrawal or protocol deviation and its reason will be recorded, and all data collected up to that point will be included in the analysis.

Adverse events (AEs) will be systematically monitored throughout the study. The research team will do regular check-ins and residents are observed by nursing staff. Additionally, all participants are free to report symptoms at any time via phone or email to the research team. Documentation will be completed by a trained member of the research team using the designated reporting form. All AEs will be assessed in terms of severity, duration, and their potential relationship to the study intervention. Depending on the nature of the event, appropriate actions will be taken by the study team, such as recommending a medical consultation, adjusting the intervention, or discontinuing study participation if necessary. Serious adverse events (SAEs) will be reported within 24 h of detection or notification. Final assessment, decisions regarding further actions, and compliance with reporting requirements lie with the study management. If needed, an additional medical evaluation will be obtained. All steps will be taken in accordance with applicable regulatory standards and Good Clinical Practice (GCP).

A formal Data Monitoring Committee is not established for this feasibility trial, as the study involves a low-risk, non-invasive intervention and a small sample size. Oversight of trial progress, safety and data integrity will be conducted internally by the research team. Any serious adverse events or ethical concerns will be immediately reported to the principal investigator and the responsible ethics committee. Also, no interim analyses, auditing procedures or specific post-trial care are planned. In the event of serious adverse events or significant implementation barriers, the project supervisor may decide to suspend or terminate the study early. Participants are covered by the institution's general liability insurance in case of harm.

## Footnotes

^1^In this study, the term ‘nursing staff’ refers to professionally trained members of the long-term care team who are directly involved in residents’ daily nursing care. In the German long-term care context, this includes registered nurses as well as trained nursing assistants. Allied health professionals who are not involved in routine nursing care are not included in this definition

## Appendix

1. TIDier Checklist
2. SPIRIT Outcomes 2022 Checklist (for combined completion of SPIRIT 2013 and SPIRIT Outcomes 2022 items)

## References

[1] United Nations Department of Economic and Social Affairs PD. World Population Ageing 2020 Highlights: Living Arrangements of Older Persons. New York: United Nations (2020). Report No.: ST/ESA/SER.A/451.

[2] Organization WH. Nursing and midwifery 2024. Available online at: https://www.who.int/news-room/fact-sheets/detail/nursing-and-midwifery (accessed August 06, 2025).

[3] White EM Aiken LH McHugh MD. Registered nurse burnout, job dissatisfaction, and missed care in nursing homes. J Am Geriatr Soc. (2019) 67(10):2065–71. 10.1111/jgs.16051 31334567 PMC6800779

[4] Jutkowitz E Landsteiner A Ratner E Shippee T Madrigal C Ullman K. Effects of nurse staffing on resident outcomes in nursing homes: a systematic review. J Am Med Dir Assoc. (2023) 24(1):75–81.e11. 10.1016/j.jamda.2022.11.002 36470321

[5] Edemekong PF Bomgaars DL Sukumaran S Schoo C. Activities of Daily Living. StatPearls. Treasure Island (FL): StatPearls Publishing (2025). Copyright © 2025, StatPearls Publishing LLC. 29261878

[6] Tatineny P Shafi F Gohar A Bhat A. Sleep in the elderly. Mo Med. (2020) 117(5):490–5. PMID: 33311760; PMCID: PMC7723148. 33311760 PMC7723148

[7] Zhu X Hu Z Nie Y Zhu T Chiwanda Kaminga A Yu Y. The prevalence of poor sleep quality and associated risk factors among Chinese elderly adults in nursing homes: a cross-sectional study. PLoS One. (2020) 15(5):e0232834. 10.1371/journal.pone.0232834 32413064 PMC7228093

[8] Prins AJ Scherder EJA van Straten A Zwaagstra Y Milders MV. Sensory stimulation for nursing-home residents: systematic review and meta-analysis of its effects on sleep quality and rest-activity rhythm in dementia. Dement Geriatr Cogn Disord. (2020) 49(3):219–34. 10.1159/000509433 32920562

[9] Lapane KL Lim E McPhillips E Barooah A Yuan Y Dube CE. Health effects of loneliness and social isolation in older adults living in congregate long term care settings: a systematic review of quantitative and qualitative evidence. Arch Gerontol Geriatr. (2022) 102:104728. 10.1016/j.archger.2022.104728 35597183 PMC12102736

[10] Bridges J Griffiths P Oliver E Pickering RM. Hospital nurse staffing and staff-patient interactions: an observational study. BMJ Qual Saf. (2019) 28(9):706–13. 10.1136/bmjqs-2018-008948 30918050 PMC6820291

[11] Allande-Cusso R Fernandez-Garcia E Porcel-Galvez AM. Defining and characterising the nurse-patient relationship: a concept analysis. Nurs Ethics. (2022) 29(2):462–84. 10.1177/09697330211046651 34879785

[12] Mok E Chiu PC. Nurse-patient relationships in palliative care. J Adv Nurs. (2004) 48(5):475–83. 10.1111/j.1365-2648.2004.03230.x 15533085

[13] Dinc L Gastmans C. Trust in nurse-patient relationships: a literature review. Nurs Ethics. (2013) 20(5):501–16. 10.1177/0969733012468463 23426234

[14] Berg L Danielson E. Patients’ and nurses’ experiences of the caring relationship in hospital: an aware striving for trust. Scand J Caring Sci. (2007) 21(4):500–6. 10.1111/j.1471-6712.2007.00497.x 18036013

[15] Park BJ Tsunetsugu Y Kasetani T Kagawa T Miyazaki Y. The physiological effects of Shinrin-yoku (taking in the forest atmosphere or forest bathing): evidence from field experiments in 24 forests across Japan. Environ Health Prev Med. (2010) 15(1):18–26. 10.1007/s12199-009-0086-9 19568835 PMC2793346

[16] Antonelli M Donelli D Carlone L Maggini V Firenzuoli F Bedeschi E. Effects of forest bathing (Shinrin-yoku) on individual well-being: an umbrella review. Int J Environ Health Res. (2022) 32(8):1842–67. 10.1080/09603123.2021.1919293 33910423

[17] Taylor E Robertson N Lightfoot C Smith A Jones C. Nature-based interventions for psychological wellbeing in long-term conditions: a systematic review. Int J Environ Res Public Health. (2022) 19:3214. 10.3390/ijerph19063214 35328901 PMC8954238

[18] Stier-Jarmer M Throner V Kirschneck M Immich G Frisch D Schuh A. The psychological and physical effects of forests on human health: a systematic review of systematic reviews and meta-analyses. Int J Environ Res Public Health. (2021) 18(4):1770. 10.3390/ijerph18041770 33670337 PMC7918603

[19] Song C Ikei H Miyazaki Y. Physiological effects of nature therapy: a review of the research in Japan. Int J Environ Res Public Health. (2016) 13(8):781. 10.3390/ijerph13080781 27527193 PMC4997467

[20] Lee I Choi H Bang KS Kim S Song M Lee B. Effects of forest therapy on depressive symptoms among adults: a systematic review. Int J Environ Res Public Health. (2017) 14(3):321. 10.3390/ijerph14030321 28335541 PMC5369157

[21] Blakeslee SB Koch AK Schröter M Jeitler M Ngandeu Schepanski S Boujnah H. Effects of greenspace interventions on mental disorders—a systematic review and meta-analysis. J Environ Psychol. (2026) 111:102919. 10.1016/j.jenvp.2026.102919

[22] Bettmann JE Speelman E Jolley A Casucci T. A systematic review and meta-analysis on the effect of nature exposure dose on adults with mental illness. Behav Sci (Basel). (2025) 15(2):153. 10.3390/bs15020153 40001784 PMC11851813

[23] Antonelli M Barbieri G Donelli D. Effects of forest bathing (Shinrin-yoku) on levels of cortisol as a stress biomarker: a systematic review and meta-analysis. Int J Biometeorol. (2019) 63(8):1117–34. 10.1007/s00484-019-01717-x 31001682

[24] Kaplan S. The restorative benefits of nature: toward an integrative framework. J Environ Psychol. (1995) 15(3):169–82. 10.1016/0272-4944(95)90001-2

[25] McDonnell AS Strayer DL. The influence of a walk in nature on human resting brain activity: a randomized controlled trial. Sci Rep. (2024) 14(1):27253. 10.1038/s41598-024-78508-x 39516236 PMC11549482

[26] Ikei H Song C Miyazaki Y. Physiological effect of olfactory stimulation by Hinoki cypress (Chamaecyparis obtusa) leaf oil. J Physiol Anthropol. (2015) 34:44. 10.1186/s40101-015-0082-2 26694076 PMC4687359

[27] Corazon S Gramkow M Poulsen D Lygum VL Zhang G Stigsdotter U. I would really like to visit the forest, but it is just too difficult: a qualitative study on mobility disability and green spaces. Scand J Disabil Res. (2019) 20:1–13. 10.16993/sjdr.50

[28] Jones R Tarter R Ross AM. Greenspace interventions, stress and cortisol: a scoping review. Int J Environ Res Public Health. (2021) 18(6):2802. 10.3390/ijerph18062802 33801917 PMC8001092

[29] Jo H Song C Miyazaki Y. Physiological benefits of viewing nature: a systematic review of indoor experiments. Int J Environ Res Public Health. (2019) 16(23):4739. 10.3390/ijerph16234739 31783531 PMC6926748

[30] van Houwelingen-Snippe J van Rompay TJL Ben Allouch S. Feeling connected after experiencing digital nature: a survey study. Int J Environ Res Public Health. (2020) 17(18):6879. 10.3390/ijerph17186879 32967093 PMC7559801

[31] Wieczorek A Schrank F Renner KH Wagner M. Psychological and physiological health outcomes of virtual reality-based mindfulness interventions: a systematic review and evidence mapping of empirical studies. Digit Health. (2024) 10:20552076241272604. 10.1177/20552076241272604 39484656 PMC11526413

[32] Riches S Jeyarajaguru P Taylor L Fialho C Little J Ahmed L. Virtual reality relaxation for people with mental health conditions: a systematic review. Soc Psychiatry Psychiatr Epidemiol. (2023) 58(7):989–1007. 10.1007/s00127-022-02417-5 36658261 PMC9852806

[33] Azizi-Zeinalhajlou A Mirghafourvand M Nadrian H Samei Sis S Matlabi H. The contribution of social isolation and loneliness to sleep disturbances among older adults: a systematic review. Sleep Biol Rhythms. (2022) 20(2):153–63. 10.1007/s41105-022-00380-x 38469248 PMC10900038

[34] Ekstrom H Svensson M Elmståhl S Sandin Wranker L. The association between loneliness, social isolation, and sleep disturbances in older adults: a follow-up study from the Swedish good aging in Skåne project. SAGE Open Med. (2024) 12. 10.1177/20503121231222823 38249948 PMC10798090

[35] Choi H Irwin MR Cho HJ. Impact of social isolation on behavioral health in elderly: systematic review. World J Psychiatry. (2015) 5(4):432–8. 10.5498/wjp.v5.i4.432 26740935 PMC4694557

[36] Chou C-C Lin J-N Yu C-P Chen C-W Yeh A-Y Lin Y-K. Effects of a virtual reality–based natural environment intervention on attention and mood in community-dwelling older adults: randomized controlled trial. JMIR Aging. (2026) 9:e87861. 10.2196/87861 41740958 PMC12980055

[37] Van Houwelingen-Snippe J Ben Allouch S Van Rompay TJL. Virtual reality representations of nature to improve well-being amongst older adults: a rapid review. J Technol Behav Sci. (2021) 6(3):464–85. 10.1007/s41347-021-00195-6 33688575 PMC7934124

[38] Alele F Malau-Aduli B. An Introduction to Research Methods for Undergraduate Health Profession Students. Townsville, QLD, Australia: James Cook University Pressbooks (2023).

[39] Kuckartz U. Mixed Methods. Methodologie, Forschungsdesigns und Analyseverfahren. Wiesbaden: Springer Fachmedien (2014).

[40] Patton M. Qualitative Research and Evaluation Methods. 4 ed Thousand Oaks: Sage Publications (2015).

[41] Brinkmann S Kvale S. InterViews: Learning the Craft of Qualitative Research Interviewing. 3 ed Thousand Oaks, CA: SAGE Publications (2015). p. 424.

[42] Buysse DJ Reynolds CF 3rd Monk TH Berman SR Kupfer DJ. The Pittsburgh sleep quality index: a new instrument for psychiatric practice and research. Psychiatry Res. (1989) 28(2):193–213. 10.1016/0165-1781(89)90047-4 2748771

[43] Hinz A Glaesmer H Brähler E Löffler M Engel C Enzenbach C. Sleep quality in the general population: psychometric properties of the Pittsburgh sleep quality index, derived from a German community sample of 9284 people. Sleep Med. (2017) 30:57–63. 10.1016/j.sleep.2016.03.008 28215264

[44] Topp CW Østergaard SD Søndergaard S Bech P. The WHO-5 well-being index: a systematic review of the literature. Psychother Psychosom. (2015) 84(3):167–76. 10.1159/000376585 25831962

[45] Brähler E Muehlan H Albani C Schmidt S. Teststatistische Prüfung und Normierung der deutschen Versionen des EUROHIS-QOL Lebensqualität-Index und des WHO-5 Wohlbefindens-Index. [Testing and standardization of the German version of the EUROHIS-QOL and WHO-5 quality-of life-indices]. Diagnostica. (2007) 53:83–96. 10.1026/0012-1924.53.2.83

[46] Russell D Peplau LA Ferguson ML. Developing a measure of loneliness. J Pers Assess. (1978) 42(3):290–4. 10.1207/s15327752jpa4203_11 660402

[47] Hudiyana J Lincoln TM Hartanto S Shadiqi MA Milla MN Muluk H. How universal is a construct of loneliness? Measurement invariance of the UCLA loneliness scale in Indonesia, Germany, and the United States. Assessment. (2022) 29(8):1795–805. 10.1177/10731911211034564 34301150

[48] Zigmond AS Snaith RP. The hospital anxiety and depression scale. Acta Psychiatr Scand. (1983) 67(6):361–70. 10.1111/j.1600-0447.1983.tb09716.x 6880820

[49] Herrmann C. International experiences with the hospital anxiety and depression scale-a review of validation data and clinical results. J Psychosom Res. (1997) 42(1):17–41. 10.1016/S0022-3999(96)00216-4 9055211

[50] Radwin LE Cabral HJ. Trust in nurses scale: construct validity and internal reliability evaluation. J Adv Nurs. (2010) 66(3):683–9. 10.1111/j.1365-2648.2009.05168.x 20423403

[51] Beaton DE Bombardier C Guillemin F Ferraz MB. Guidelines for the process of cross-cultural adaptation of self-report measures. Spine (Phila Pa 1976). (2000) 25(24):3186–91. 10.1097/00007632-200012150-00014 11124735

[52] Kuckartz U Rädiker S. Qualitative inhaltsanalyse. In: Methoden, Praxis, Computerunterstützung. 6. Auflage ed. Weinheim: Beltz Juventa (2024).

[53] VERBI Software. MAXQDA 2025. Berlin: VERBI Software (2025).

[54] IBM Corporation. IBM SPSS Statistics for Windows. Version 270. Armonk (NY): IBM Corporation (2020).

[55] Yuen I Kwok T. Effect of virtual zen garden on quality of life of residents in long-term care home. Int J Environ Res Public Health. (2025) 22(4):510. 10.3390/ijerph22040510 40283736 PMC12027189

[56] Hayden L Chaze F Kamath A Azevedo A Bucko D Jackson A. Implementation of a virtual reality recreation program in long-term care. J Rehabil Assist Technol Eng. (2022) 9:205566832110709. 10.1177/20556683211070994 PMC8905195 35281782

[57] Khirallah Abd El Fatah N Abdelwahab Khedr M Alshammari M Mabrouk Abdelaziz Elgarhy S. Effect of immersive virtual reality reminiscence versus traditional reminiscence therapy on cognitive function and psychological well-being among older adults in assisted living facilities: a randomized controlled trial. Geriatr Nurs (Minneap). (2024) 55:191–203. 10.1016/j.gerinurse.2023.11.010 38007908

[58] Souchet AD Lourdeaux D Burkhardt J-M Hancock PA. Design guidelines for limiting and eliminating virtual reality-induced symptoms and effects at work: a comprehensive, factor-oriented review. Front Psychol. (2023) 14:1–32. 10.3389/fpsyg.2023.1161932 PMC10288216 37359863

[59] Appel L Kisonas E Appel E Klein J Bartlett D Rosenberg J. Administering virtual reality therapy to manage behavioral and psychological symptoms in patients with dementia admitted to an acute care hospital: results of a pilot study. JMIR Form Res. (2021) 5(2):e22406. 10.2196/22406 33533720 PMC7889418

[60] Kim JH Park S Lim H. Developing a virtual reality for people with dementia in nursing homes based on their psychological needs: a feasibility study. BMC Geriatr. (2021) 21(1):167. 10.1186/s12877-021-02125-w 33678160 PMC7938563

[61] dr. dresing & pehl GmbH. f4 (Software for Transcription & Qualitative Analysis). Marburg, Germany: dr. dresing & pehl GmbH (2025).

[62] Union EPaCotE. Regulation (EU) 2016/679 of the European parliament and of the council of 27 April 2016 on the protection of natural persons with regard to the processing of personal data and on the free movement of such data, and repealing directive 95/46/EC (General Data Protection Regulation) (2016). Available online at: https://eur-lex.europa.eu/legal-content/EN/TXT/PDF/?uri=OJ:L:2016:119:FULL (accessed August 20, 2025).

[63] Federal Republic of Germany. Federal Data Protection Act (BDSG). Translation Including Amendments by Article 10 of the Act of 23 June 2021 ed. Berlin, Germany: Federal Ministry of the Interior, Language Service (2021).

